# The characteristics of liver nodules in biliary atresia native liver survivors

**DOI:** 10.1007/s00383-026-06644-8

**Published:** 2026-09-22

**Authors:** John Yeh Joon Park, Patrick Ho Yu Chung, Fanny Yeung, Kenneth Kak Yuen Wong

**Affiliations:** Division of Paediatric Surgery, Department of Surgery, School of Clinical Medicine, Li Ka Shing Faculty of Medicine, The University of Hong Kong, Hong Kong SAR, Hong Kong

**Keywords:** Biliary atresia, Kasai portoenterostomy, Liver nodule, Paediatric

## Abstract

**Purpose:**

To delineate features of hepatic nodules in native liver survivors (NLS) of biliary atresia (BA) post-Kasai portoenterostomy (KPE) and determine the surveillance utility of tumour markers and imaging studies.

**Methods:**

This retrospective study conducted in a tertiary referral centre evaluated post-KPE patients who developed liver nodules following operations performed between 1979–2020. These included NLS at time of writing and liver transplant (LT) recipients with evidence of nodules before LT. Demographic, biochemistry, tumour markers, imaging, and histopathological data were collected. Dysplastic and non-dysplastic nodules were compared using Mann-Whitney U and Fisher’s exact test.

**Results:**

Among 163 BA patients, of 96 NLS, 17 developed nodules; of 67 post-LT patients, 6 developed nodules before LT. Together, 23 post-KPE patients with nodules were analyzed: 4 dysplastic (one progressed into early hepatocellular carcinoma) and 19 benign lesions. Dysplastic nodules appeared earlier (8.2 vs. 21.2 years, p=0.286) and were larger (11.6 vs. 2.2cm^2^, p=0.284). Alpha-fetoprotein (AFP) levels were similar (2.0ng/mL vs. 3.0ng/mL, p=0.232). All dysplastic nodules exhibited arterial-phase enhancement without portovenous washout on contrast-enhanced computed tomography (CT).

**Conclusion:**

In BA NLS, one-quarter develop hepatic nodules over time; malignant changes are rare, but can occur. Additionally, contrast-enhanced CT surveillance may be useful for larger, or earlier-detected hepatic nodules.

## Introduction

Biliary atresia (BA) is a rare, progressive sclerosing fibro-obliteration of the intra- and extrahepatic bile ducts that presents in the first few weeks of life [23]. Prompt surgical correction with Kasai portoenterostomy (KPE) after clinical and radiological diagnosis has traditionally been considered only a palliative bridge to liver transplantation (LT) – the only definitive treatment for end-stage liver disease secondary to BA.

However, emerging international data challenge this view. The largest European series report native liver survival of 41–55% at 5 years and 35–47% at 10 years after KPE [5, 7, 9, 17]. In French and Dutch national cohorts, 26% and 27% of patients survived with their native liver for at least 20 years, respectively [6, 14]. The Japanese Biliary Atresia Registry documented a 20-year native liver survival of 49% among 3160 patients [16]. Moreover, in both European and Japanese studies, younger age at KPE (particularly ≤ 30 days) was associated with significantly higher native liver survival, with a difference of up to 20% compared to those operated after 60 days. Together, these findings challenge the conventional view of KPE as merely a temporary measure and demonstrate that durable native liver survival is achievable in a substantial proportion of BA patients [25]. Nevertheless, native liver survivors (NLS) remain at risk of progressive cholestasis, liver fibrosis, and portal hypertension. Whilst these conditions have been extensively evaluated, the development of hepatic nodules after KPE remains poorly understood [3, 22].

To date, the clinical characteristics and natural history of hepatic nodules in long-term NLS after KPE are poorly defined. The spectrum of nodular lesions – ranging from regenerative nodules to dysplastic changes and potential malignancy – has not been systematically characterized in patients with BA. A recent analysis of liver explants from BA patients undergoing LT revealed that nodules were present in 43% of explanted livers, and that a quarter of these patients had premalignant or malignant lesions; notably, the majority of these nodules had not been detected by pre-transplant imaging or serum alpha-fetoprotein (AFP) levels [2]. Thus, clinically relevant nodular lesions are common in this population, yet current surveillance methods frequently fail to identify them. Moreover, the utility of serum tumor markers such as AFP and carcinoembryonic antigen (CEA) for differentiating benign from dysplastic or malignant nodules has not been established in BA patients, and standardized imaging protocols specific to post-KPE nodule surveillance and diagnosis are lacking.

The main purposes of this study were (1) to describe our hospital-wide experience in the management of hepatic nodules in NLS of BA; and (2) to present the clinical characteristics and nature of these nodules, including the distinction between dysplastic and non-dysplastic lesions; and (3) to discuss surveillance and management strategies apart from LT.

## Methods

### Study design and patients

This retrospective study included BA patients who underwent KPE between 1979 and 2020 at a single tertiary paediatric surgical center, with additional cases referred from other regional hospitals. Their data were retrieved from hospital databases, and their medical records were reviewed. This study was performed in compliance with ethical standards and the declaration of Helsinki.

### Patient selection and data extraction

Medical records were identified using ICD-9 codes: 751.61 (biliary atresia) and 51.37 (Kasai portoenterostomy). Inclusion criteria were: (1) all native liver survivors (NLS) at the time of writing whose KPE was performed between 1979 and 2020; and (2) all BA patients who underwent LT after KPE between 1979 and 2020 and had radiological documentation of hepatic nodules prior to LT. Patients were excluded if they had incomplete follow-up data, missing key laboratory values, or no imaging surveillance performed.

Among eligible subjects, we identified all patients who developed hepatic nodules during follow-up. For each such patient, extracted data included: demographic information (sex, date of birth, age at KPE), dates of KPE and of nodule detection (to calculate interval from KPE to nodule detection), liver function test and tumor marker levels at the time of hepatic nodule detection – including bilirubin, alanine aminotransferase (ALT), aspartate aminotransferase (AST), alkaline phosphatase (ALP), AFP, and CEA – diagnostic modality, histopathology results (if biopsied), endoscopic findings (presence and grading of esophageal varices, history of banding or sclerotherapy, signs of portal hypertension), and clinical history of cholangitis.

### Definitions of clinical variables and nodule classification

Hyperbilirubinemia was defined as total serum bilirubin of > 20µmol/L. Elevated ALP was defined as > 110U/L, elevated ALT as > 58U/L, and elevated AST as > 38U/L, based on the Hong Kong Hospital Authority laboratory references ranges for liver biochemistry.

Cholangitis was defined as at least one documented episode of fever (core body temperature > 38.5 °C) accompanied by a rise in bilirubin above baseline, requiring intravenous antibiotics and/or future antibiotic prophylaxis. Recurrent cholangitis was defined as two or more separate episodes.

Portal hypertension was assessed by the presence of esophageal varices (any grade) on endoscopy, splenomegaly (clinically palpable or confirmed by ultrasonography), ascites, or portal hypertensive gastropathy.

Hepatic nodules were classified based on histopathology reports when available (biopsy or explant specimen) or on a combination of characteristic imaging features and clinical follow-up. Nodules were categorised as dysplastic, regenerative, calcified granuloma, inflammatory pseudotumor, cyst, and haemangioma. Only the four dysplastic nodules were biopsy-confirmed; all other nodules received a radiological diagnosis. Dysplastic nodules were defined histologically as regenerative nodules that demonstrate cytological atypia, nuclear crowding, and architectural distortion, accompanied by a variable number of unpaired arterioles or capillaries, in the absence of definitive malignant features.

### Statistical analysis

Statistical analysis was performed using GraphPad Prism (version 10.0, GraphPad Software, San Diego, USA) and SPSS (version 26.0, SPSS Inc., Armonk, USA). Continuous variables were summarised as medians with interquartile ranges (IQR) as the data were not normally distributed. Comparisons between dysplastic and non-dysplastic nodule groups were performed using Mann-Whitney U test (two-tailed) for continuous variables and Fisher’s exact test for categorical variables. A p-value < 0.05 was considered statistically significant.

## Results

### Study population and patient selection

**Fig. 1:**
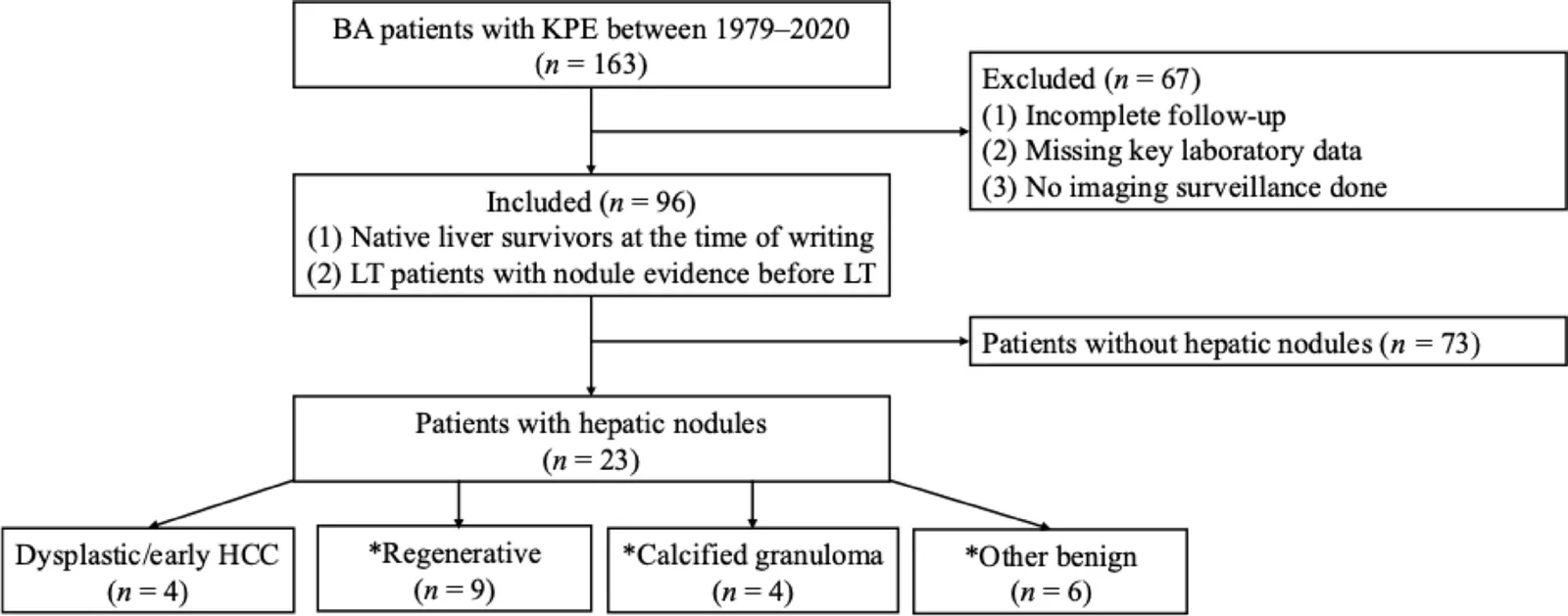
Flowchart of patient selection in this study. Among 96 patients meeting the inclusion criteria, 23 (24.0%) developed hepatic nodules: 4 dysplastic/early HCC (17.4%), 9 regenerative (39.1%), 4 calcified granulomas (17.4%), and 6 other benign lesions (26.1%). *Radiological diagnosis was used for benign lesions without histopathology.* BA *biliary atresia, *KPE *Kasai portoenterostomy, *LT *liver transplantation, *HCC *hepatocellular carcinoma

During the study period, a total of 163 patients with BA underwent KPE at a single tertiary paediatric surgical center in Hong Kong. After excluding 67 patients with incomplete follow-up, missing of key laboratory values, or lack of imaging surveillance, 96 patients were included in the analysis. Of these, 23 patients (24%) developed hepatic nodules (Fig. 1).

### Patient characteristics

Among those with hepatic nodules, the median age at KPE was 61 days (range: 32–152 days). The cohort comprised 11 females (47.8%) and 12 males (52.2%). The median interval from KPE to nodule detection was 12.8 years (IQR 6.5–31.8 years). Endoscopic data were available for 22 of the 23 patients. Esophageal varices were documented in 16 patients (72.7%) and portal hypertensive gastropathy in 2 patients (9.1%). Endoscopic banding or sclerotherapy was performed in 7 patients. Splenomegaly was present in 8 patients, and ascites was present in 2. The demographic and clinical characteristics of the 23 patients with hepatic nodules are summarized in Table 1.

**Table 1:** Patient characteristics of 23 native liver survivors who developed hepatic nodules post-KPE. ^1^*IQR* = interquartile range (25th − 75th percentile); ^2^One patient had no endoscopic data; percentages are based on 22 evaluable patients

Patient characteristics	Median (IQR^1^) or number (%)
*Demographics*	
Male	12 (47.8%)
Female	11 (52.2%)
Age at Kasai portoenterostomy (days)	61 (52–72)
KPE to nodule detection interval (years)	12.8 (6.5–31.8)
*Evidence of portal hypertension (n = 22 evaluable)* ^2^	
Any esophageal varices	16 (72.7%)
Grade I	6 (27.3%)
Grade II	4 (18.2%)
Grade III	4 (18.2%)
Unknown grade	2 (9.1%)
Banding or sclerotherapy	7 (31.8%)
Portal hypertensive gastropathy	2 (9.1%)
Splenomegaly	8 (36.4%)
Ascites	2 (9.1%)
*Cholangitis*	
Any	14 (60.9%)
Recurrent	11 (47.8%)

### Nodule characteristics and management

Upon detection of a hepatic nodule, all patients underwent blood tests including liver function tests (bilirubin, ALT, AST, ALP) and tumour markers (AFP, CEA). Ultrasonography was the initial imaging modality. If a nodule was identified, contrast-enhanced computed tomography (CT) was performed for further characterisation. Lesions suspicious on contrast-enhanced CT were further evaluated with magnetic resonance imaging (MRI) or positron emission tomography-CT (PET-CT). Biopsy was reserved exclusively for cases with radiological evidence of dysplasia or malignant change; consequently, only the four dysplastic nodules underwent histopathological confirmation. All other nodules received a radiological diagnosis based on imaging characteristics.

Contrast-enhanced CT was the primary diagnostic imaging modality in nine patients (39.1%), followed by ultrasonography in seven (30.4%). The remaining nodules were diagnosed by MRI or PET-CT. No benign nodules underwent confirmatory biopsies.

**Fig. 2:**
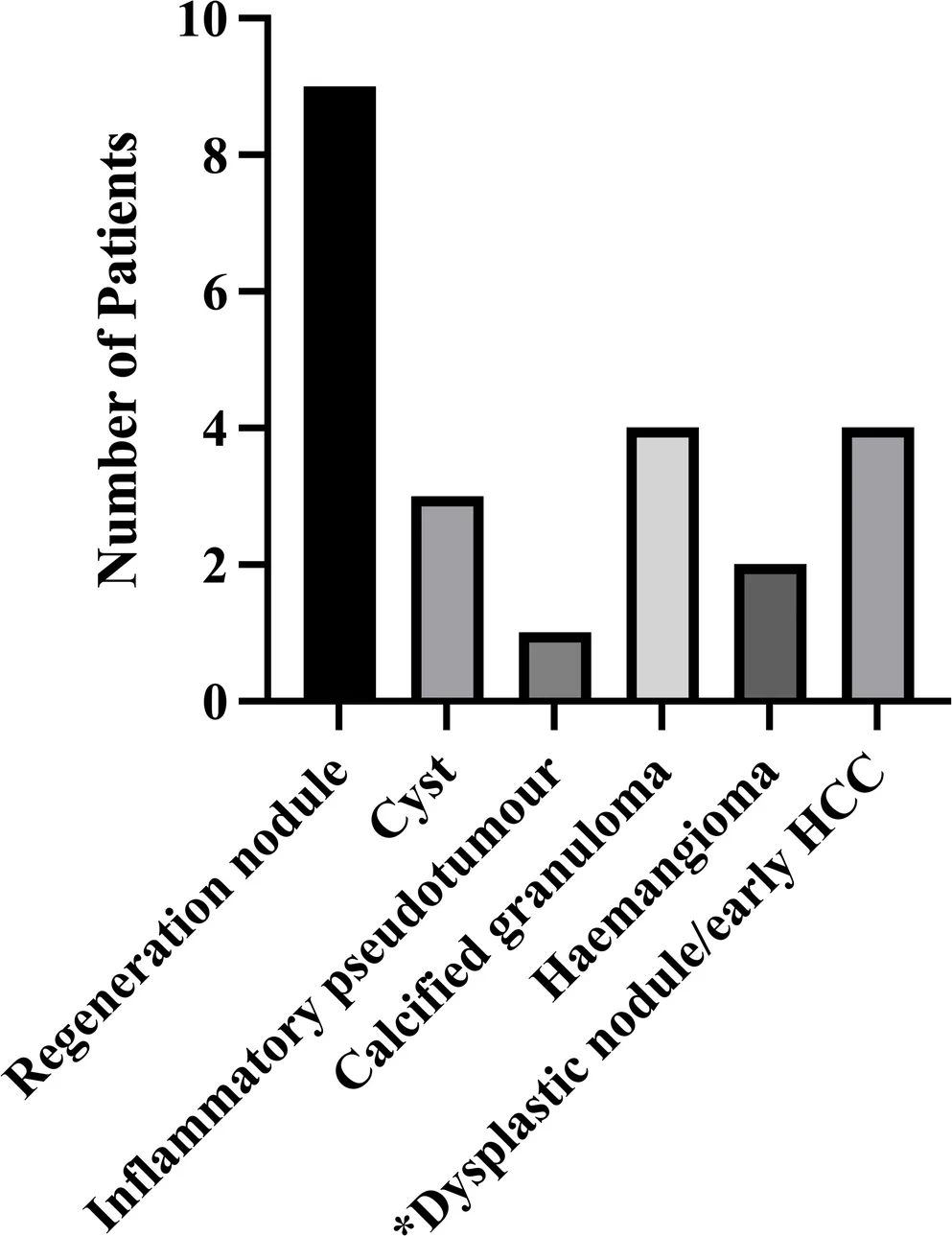
Distribution of hepatic nodule subtypes among 23 NLS of BA. *Biopsy proven

Regenerative nodules were the most frequent lesion, occurring in nine patients (39.1%). Four patients had biopsy-proven dysplastic nodules (17.4%); one of these later progressed to a well-differentiated hepatocellular carcinoma (HCC), confirmed on liver explant. Calcified granulomas were found in another four patients (17.4%), and the remaining six patients (26.1%) had other benign nodules (Fig. 2) A detailed breakdown of nodule types, diagnostic modalities, and biochemical abnormalities is presented in Table 2.

**Table 2:** Characteristics of hepatic nodules in 23 post-KPE BA patients. 1AST data available for 21 patients; two patients missing

Characteristic	Number (%)
*Radiological diagnosis of nodule*	
HCC	1 (4.3%)
Dysplastic	3 (13.0%)
Regenerative	9 (39.1%)
Calcified granuloma	4 (17.4%)
Other benign	6 (26.1%)
*Primary diagnostic modality*	
Contrast-enhanced CT	9 (39.1%)
Ultrasonography	7 (30.4%)
Others (MRI, PET-CT, biopsy)	7 (30.4%)
*Liver biochemistry abnormalities*	
Hyperbilirubinemia (> 23µmol/L)	19 (82.6%)
Elevated ALP (> 110U/L)	22 (95.7%)
Elevated ALT (> 58U/L)	14 (60.9%)
Elevated AST (> 38U/L)	18^1^ (85.7%)

At the time of nodule diagnosis, biochemical evidence of impaired liver function was common. Persistent hyperbilirubinemia was present in 19 patients (82.6%). Elevated ALP was observed in 22 patients (95.7%), elevated ALT in 14 (60.9%), and elevated AST in 18 of 21 patients with available data (85.7%).

### Age at KPE and KPE-to-nodule detection interval

**Fig. 3:**
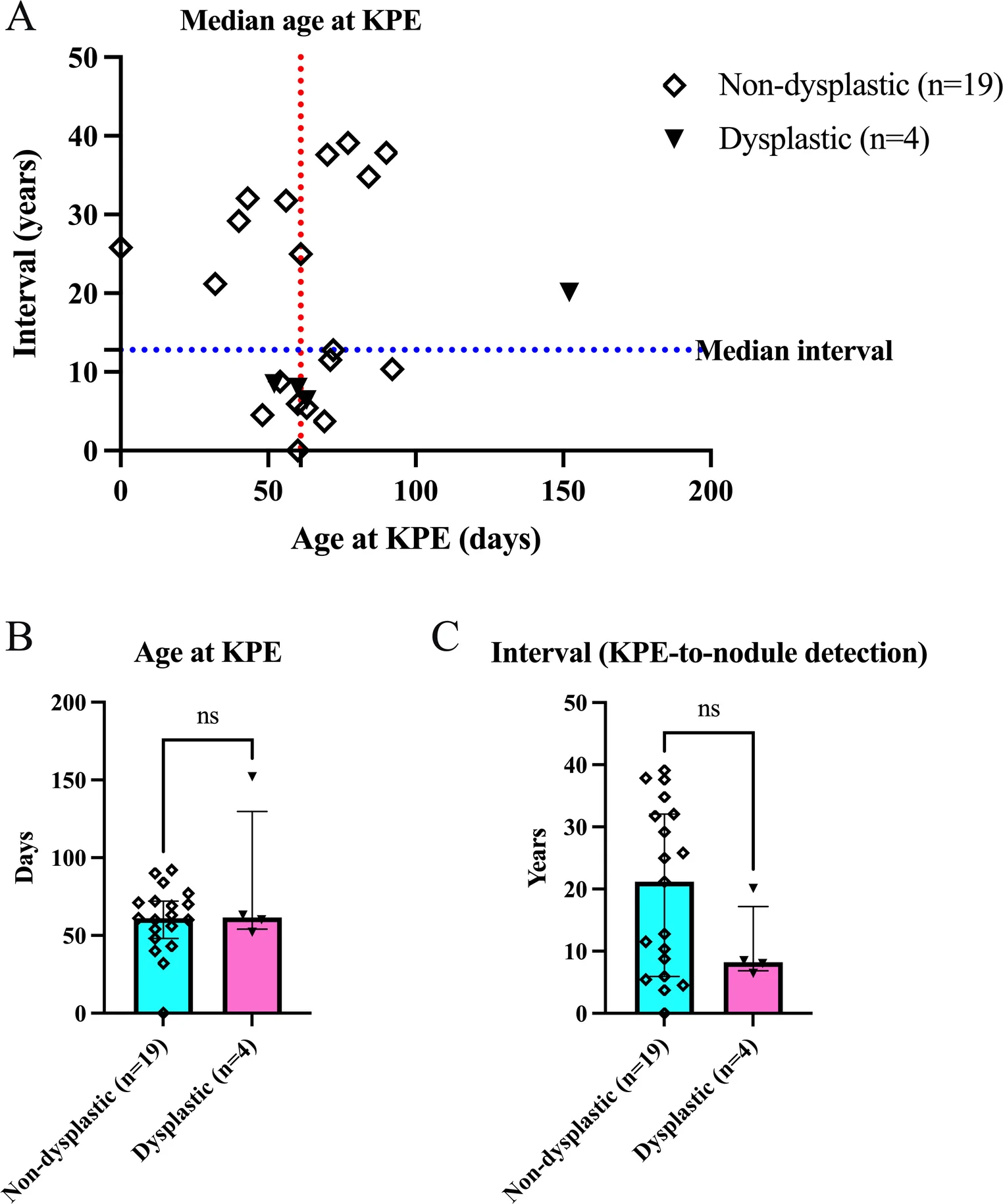
Comparison of age at KPE and interval between non-dysplastic (n=19, ◇) and dysplastic (n=4, ▼) groups. **a** Scatter plot showing individual patient data: age at KPE (days) on x-axis and interval (years) on y-axis. Red dashed line (…) represents median age at KPE; blue dashed line (…) represents median interval. **b** Bar graph comparing median age at KPE (p=0.734, Mann-Whitney U test). **c** Bar graph comparing median interval (p=0.286, Mann-Whitney U test). *ns *not significant; *KPE *Kasai portoenterostomy

We compared age at KPE and the interval from KPE-to-nodule detection between the dysplastic and non-dysplastic groups (Fig. 3a). The median age at KPE was similar between groups (61.5 days vs. 61.0 days; Fig. 3b). For the interval from KPE-to-nodule detection, the dysplastic group had a shorter median interval than the non-dysplastic group (8.2 years vs. 21.2 years), but the difference was not statistically significant (Fig. 3c).

### Comparing dysplastic and non-dysplastic nodules

**Fig. 4:**
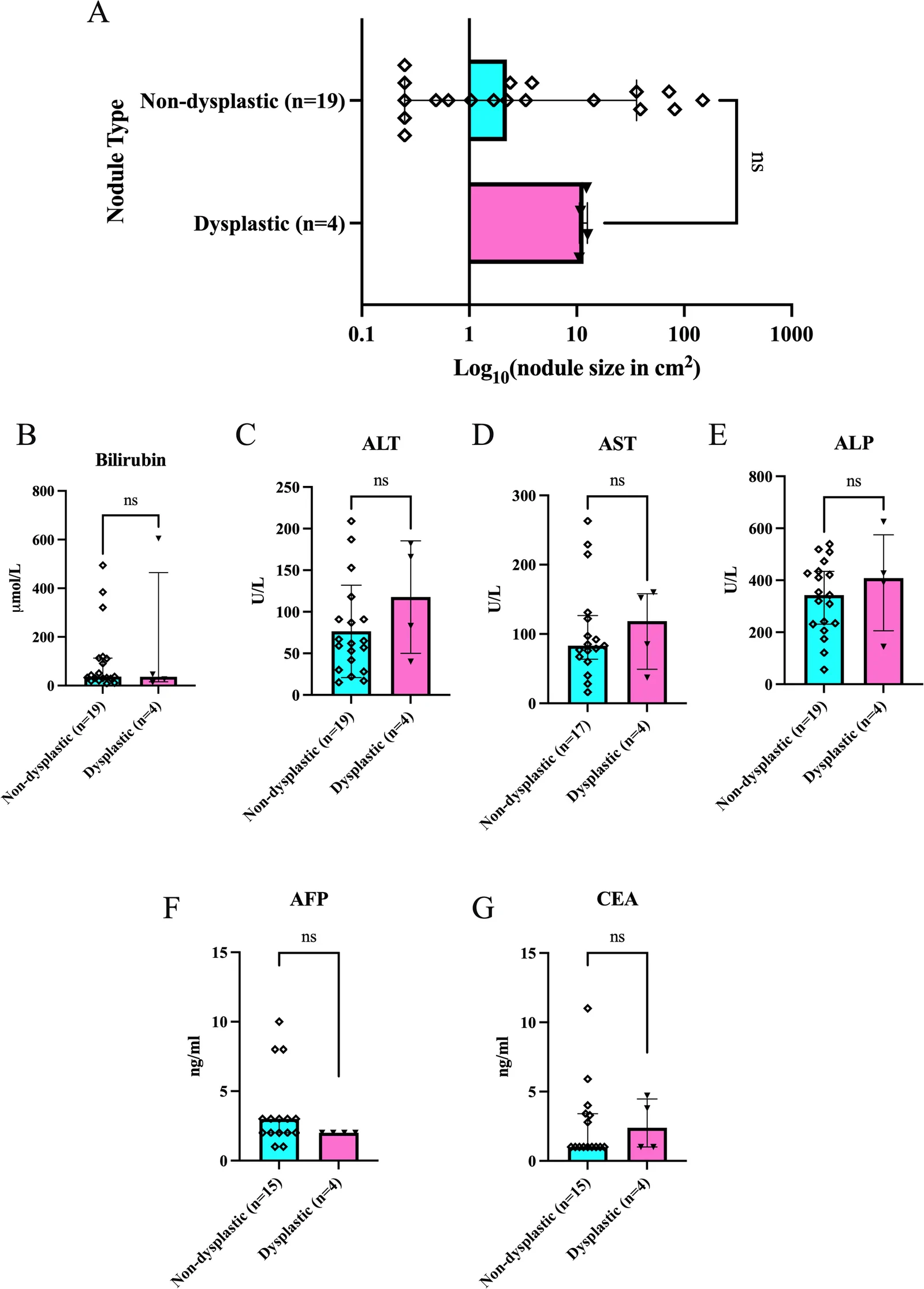
Comparison of clinical and biochemical parameters between non-dysplastic (n=19, ◇) and dysplastic (n=4, ▼) nodule groups. All p-values are from two-tailed Mann-Whitney U tests. **a** Box plot of nodule size on a log_10_ scale, p=0.284; **b** Box plot of serum bilirubin, p=0.907; **c** Box plot of ALT, p=0.318; **d** Box plot of AST, data available from 21 patients, p=0.646; **e** Box plot of ALP, p=0.611; **f** Box plot of AFP, data available from 15 non-dysplastic and 4 dysplastic patients, p=0.232; **g** Box plot of CEA, data available from 15 non-dysplastic and 4 dysplastic patients, p=0.730. *ns* not significant

A comparison of clinical and biochemical parameters between the non-dysplastic and dysplastic nodules was made. Nodule size was larger in the dysplastic group (median 11.6cm^2^, IQR 10.6–12.5) than in the non-dysplastic group (median 2.24cm^2^, IQR 0.25–36.0). However, the difference did not reach statistical significance (Fig. 4a).

Bilirubin and serum liver enzyme levels on nodule diagnosis were also compared. Median bilirubin was 37µmol/L (IQR 29–113) in non-dysplastic versus 36µmol/L (IQR 16–465) in dysplastic nodules. Median ALT was 62U/L (IQR 30–91) versus 125U/L (IQR 51–178); median AST was 83U/L (IQR 64–127) versus 119U/L (IQR 49–158); and median ALP was 343U/L (IQR 231–434) versus 409U/L (IQR 206–575) (Fig. 4b, c, d, e).

Tumour marker levels were also similar between groups. Median AFP was 3.0 ng/ml (IQR 1.0–3.0) in non-dysplastic and 2.0 ng/ml (IQR 2.0–2.0) in dysplastic nodules. Median CEA was 1.0 ng/ml (IQR 1.0–3.4) in non-dysplastic compared with 2.4 ng/ml (IQR 1.0–4.3) in dysplastic nodules. AFP and CEA data were available for 15 non-dysplastic and all 4 dysplastic patients (Fig. 4f, g).

Additionally, a history of at least one episode of cholangitis after KPE was documented in 14 of the 23 patients (60.9%). Recurrent cholangitis was present in 11 patients (47.8%). Among the 14 patients with any cholangitis, recurrent episodes were documented in all three dysplastic patients and in 8 of 11 non-dysplastic patients. The full-cohort comparisons are summarised in Table 3.

**Table 3:** Clinical and biochemical characteristics of hepatic nodules in 23 post-KPE BA patients. ^1^AST data available for 17 non-dysplastic patients (two missing) and 4 dysplastic patients; ^2^AFP/CEA data available for 15 non-dysplastic (four missing) and 4 dysplastic patients; ^3^Mann-Whitney U test (two-tailed); *p* < 0.05 as statistically significant; ^4^Fisher’s exact test (two-tailed): odds ratio not calculated due to small sample size

**Characteristic**	**Non-dysplastic** (*n* = 19)	**Dysplastic** (*n* = 4)	**p-value**	
Nodule size (cm^2^)	2.2 (0.3–36.0)	11.6 (10.6–12.5)	0.284^3^	
Bilirubin (µmol/L)	37 (29–113)	36 (16–465)	0.907^3^	
ALT (U/L)	62 (30–91)	125 (51–178)	0.318^3^	
AST (U/L)^1^	83 (64–127)	119 (49–158)	0.646^3^	
ALP (U/L)	343 (231–434)	409 (206–575)	0.611^3^	
AFP (ng/ml)^2^	3.0 (1.0–3.0)	2.0 (2.0–2.0)	0.232^3^	
CEA (ng/ml)^2^	1.0 (1.0–3.4)	2.4 (1.0–4.5)	0.730^3^	
Any cholangitis, n (%)	11 (57.9)	3 (75.0)	> 0.999^4^	
Recurrent cholangitis, n (%)	8 (42.1)	3 (75.0)	0.317^4^	

### Imaging features of dysplastic nodules

**Fig. 5:**
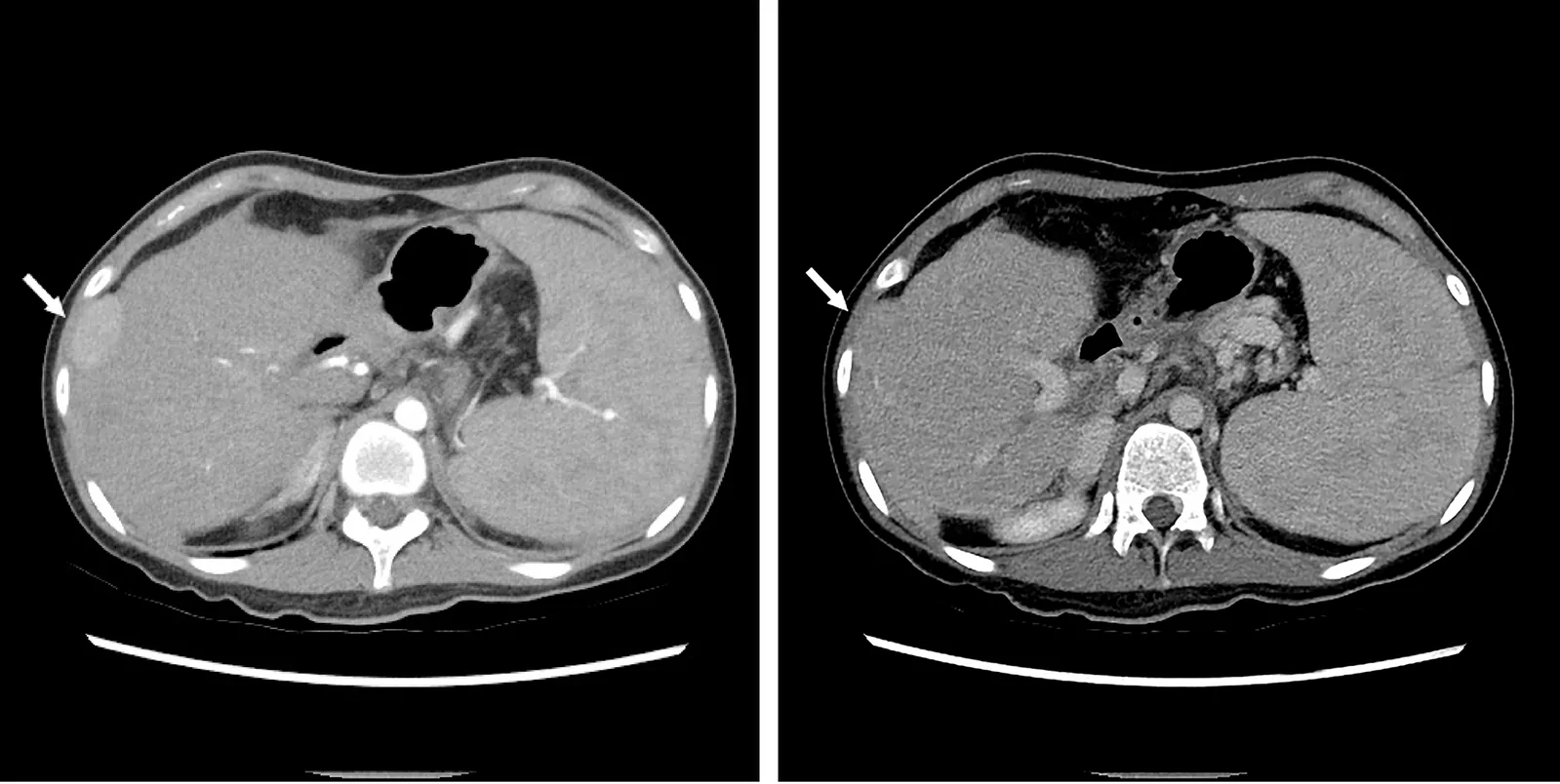
Representative images of dysplastic nodules in BA NLS; nodules show arterial phase enhancement (left) and lack of portovenous phase washout (right). The solitary nodule is indicated with an arrow (➨)

The four dysplastic nodules were diagnosed with a triphasic CT scan, followed by a confirmatory biopsy. All dysplastic nodules exhibited arterial phase enhancement. However, definitive portovenous washout was not observed in any nodule, although one nodule showed faint delayed washout in larger lesions. This nodule later developed into a well-differentiated HCC. The detailed CT characteristics of each nodule are summarized in Table 4, and representative images are shown (Fig. 5).

**Table 4:** Contrast-enhanced CT findings of the four dysplastic hepatic nodules on diagnosis. All nodules demonstrated arterial phase enhancement. Portovenous washout was absent or equivocal in most; none showed definite washout. Sizes are given as maximum dimensions. *PV* portovenous phase

Nodule	Size (cm)	Arterial phase	PV phase	Other features
#1	3.3 × 3.2 × 4.0 (segment IVb)	Arterial enhancing	Faint delayed washout in larger lesions; mostly isodense	Central hypodensity
#2	3.5 × 1.9 × 2.8 (subcapsular right lobe)	Arterial enhancing	Hypodense on PV phase	Persistent arterial enhancing lesions 0.5 cm in segments VIII and IV (isodense in PV)
#3	3.5 (segment VIII) + ill-defined exophytic lesion	Arterial enhancing	No contrast washout (for 3.5 cm lesion); suspicious washout in exophytic lesion	1.5 cm hypodense left lateral segment lesion
#4	1.6 × 2.1 × 1.8 (segment IVa/VIII)	Faint arterial enhancing	No contrast washout (isodense in PV and delayed phase)	–

## Discussion

In this retrospective study of native liver survivors (follow-up ranging from 1.3 to 39.2 years, median 21.3 years) after KPE, we found that approximately one-quarter of patients develop hepatic nodules. Dysplastic changes were confirmed in 17%, with one patient’s nodule eventually developing into a well-differentiated early HCC. These figures are in line with previous reports that NLS of BA are at risk of developing nodular lesions, but that malignant transformation and carcinoma remain rare [24, 26]. Nevertheless, the presence of biopsy-proven HCC and dysplastic nodules indicate that premalignant and malignant lesions do occur in this population and warrant systematic surveillance.

The clinical characteristics of the 23 patients with nodules showed a median age at KPE of 61 days and a median interval of KPE-to-nodule detection of 12.8 years. Esophageal varices were present in nearly three-quarters of those who underwent endoscopy, and a history of cholangitis was documented in 61%. These findings highlight that these NLS frequently exhibit signs of portal hypertension and recurrent biliary tract infection – known drivers of liver fibrosis – hence creating a microenvironment conducive to dysplastic changes [4].

When we compared the 19 non-dysplastic with the 4 dysplastic nodules, several observations emerged, although most differences did not reach statistical significance – largely because of the very small number of dysplastic cases (*n* = 4). The median age at KPE was almost identical between the two groups (61.5 days versus 61.0 days; *p* = 0.734). This suggests that the timing of surgery does not predict the risk of dysplastic transformation; other factors – such as ongoing cholestatic injury, genetic susceptibility, or postoperative complications – may be more relevant [8, 13, 19].

By contrast, the interval from KPE-to-nodule detection was markedly shorter in the dysplastic group (median 8.2 years versus 21.2 years), although the difference was not statistically significant (*p* = 0.286). The trend is biologically plausible: dysplastic lesions, as potential precursors of malignancy including HCC, may develop more rapidly under chronic inflammatory and cholestatic stress [1, 12]. The wide overlap in individual values and the small number of dysplastic nodules, however, preclude a firm conclusion; nevertheless, the observation that dysplastic nodules tended to appear earlier suggests that when a hepatic nodule is detected relatively soon after KPE, it should raise clinical suspicion for dysplasia. Larger multicenter studies are needed to confirm this association.

Dysplastic nodules also tended to be larger (11.56 cm^2^ vs. 2.24 cm^2^; *p* = 0.284). Importantly, some benign regenerative nodules were also large (up to 148.75 cm^2^), so size alone is an unreliable discriminator [10, 20]. More valuably, all four biopsy-proven dysplastic nodules demonstrated a consistent imaging pattern on contrast-enhanced CT: arterial phase enhancement without definite portovenous washout. This pattern differs from simple cysts (non-enhancing) and regenerative nodules (rarely shows persistent arterial enhancement), suggesting that contrast-enhanced CT is highly useful for characterization.

In contrast, serum biochemistry and tumor markers were of little discriminatory value. The median levels of latest follow-up bilirubin, ALT, AST, and ALP did not differ significantly between groups. More importantly, median AFP and CEA levels of diagnosis were almost identical between dysplastic and non-dysplastic nodules (AFP 2.0 ng/mL in both; CEA 1.0 vs. 2.4 ng/mL; *p* = 0.232 and 0.730, respectively). These results suggest that AFP may not be a reliable biomarker for dysplasia in this small cohort, but the limited size and incomplete data preclude a definitive conclusion. Larger, prospective studies are needed to assess the utility of AFP and other tumour markers in BA NLS. Our findings suggest that AFP is not a reliable biomarker for dysplasia in this population, and reliance on AFP or CEA alone for surveillance would be inadequate [15, 18].

A recent review on paediatric Liver Imaging Reporting and Data System (LI-RADS) notes that for BA, the observed nodules include regenerative, dysplastic, focal nodular hyperplasia-like lesions, and HCC, but no established surveillance protocol exists; the authors suggest ultrasound and AFP every 6–12 months [21]. Our results challenge the reliance on AFP in that suggestion, as we found no difference in AFP levels between dysplastic and benign nodules. Based on these preliminary observations, we suggest that contrast-enhanced CT may be considered more frequently in the early post-KPE period (e.g., every 1–2 years), especially when a nodule develops early, is large or rapidly growing, despite normal AFP. This approach aims to detect potentially dysplastic and malignant nodules that might otherwise be missed by relying on tumour markers alone. However, given the small number of dysplastic cases (*n* = 4), these findings should be interpreted cautiously and validated in larger, prospective cohorts.

Cholangitis is another common complication after KPE, and recurrent episodes may contribute to dysplastic transformation [11]. Among the 14 patients with any cholangitis, all three dysplastic patients had recurrent episodes (100%), compared with 8 of 11 (73%) non-dysplastic patients. The numbers were too small for statistical testing, so we report this observation descriptively. The universal occurrence of recurrent cholangitis in the dysplastic group is hypothesis-generating and suggests a possible link between repeated biliary infection and dysplasia. A larger cohort or prospective studies are needed to explore this association.

Only the four dysplastic nodules underwent biopsy; no benign nodules were biopsied. This reflects our selective biopsy policy based on suspicious imaging features. Nevertheless, in other settings where biopsy is performed more liberally, our findings support that invasive procedures may be avoided if contrast-enhanced CT findings are used as the primary diagnostic tool. In the absence of atypical imaging features, a radiological diagnosis may be sufficient. Biopsy should be reserved for lesions with atypical features such as washout, growth on serial imaging, or indeterminate morphology such as exophytic lesions.

A strength of this study is its exceptionally long follow-up period (up to 39.2 years, median 21.3 years), which provides unique insight into the natural history of hepatic nodules in BA NLS – a population for which long-term data remains scarce. The comprehensive characterisation of nodule imaging and pathology, provides a valuable framework for understanding nodule behaviour in this growing patient population – an area with very limited published data.

However, several limitations must be acknowledged. The retrospective design may introduce information bias, and the small number of dysplastic nodules (n = 4) limits statistical power. Potential bias is further increased by incomplete clinical and pathological data for some nodules, including missing AFP/CEA/AST values and the absence of biopsy confirmation for any radiologically benign nodule. Furthermore, only the four dysplastic nodules underwent histopathological confirmation; all benign lesions were diagnosed radiologically. This introduces significant diagnostic uncertainty, as some lesions classified as regenerative or benign may have contained premalignant changes that were not detected. However, all patients with benign nodules had stable imaging features on latest follow-up, which strengthens the radiological diagnosis despite the absence of biopsy.

Additionally, the study spans more than four decades (1979–2020), during which surgical techniques, imaging modalities, surveillance protocols, and transplantation indications evolved substantially. These changes may have introduced heterogeneity in patient management and outcomes. Moreover, a large number of patients (67 of 163, 41%) were excluded due to incomplete follow-up, missing laboratory values, or lack of imaging surveillance. This may have introduced selection bias, as excluded patients may have had different clinical characteristics or outcomes. Indeed, as a single-hospital study, generalisability to other populations may be limited, but the findings are likely applicable to other Asian centres with similar disease epidemiology.

**Fig. 6:**
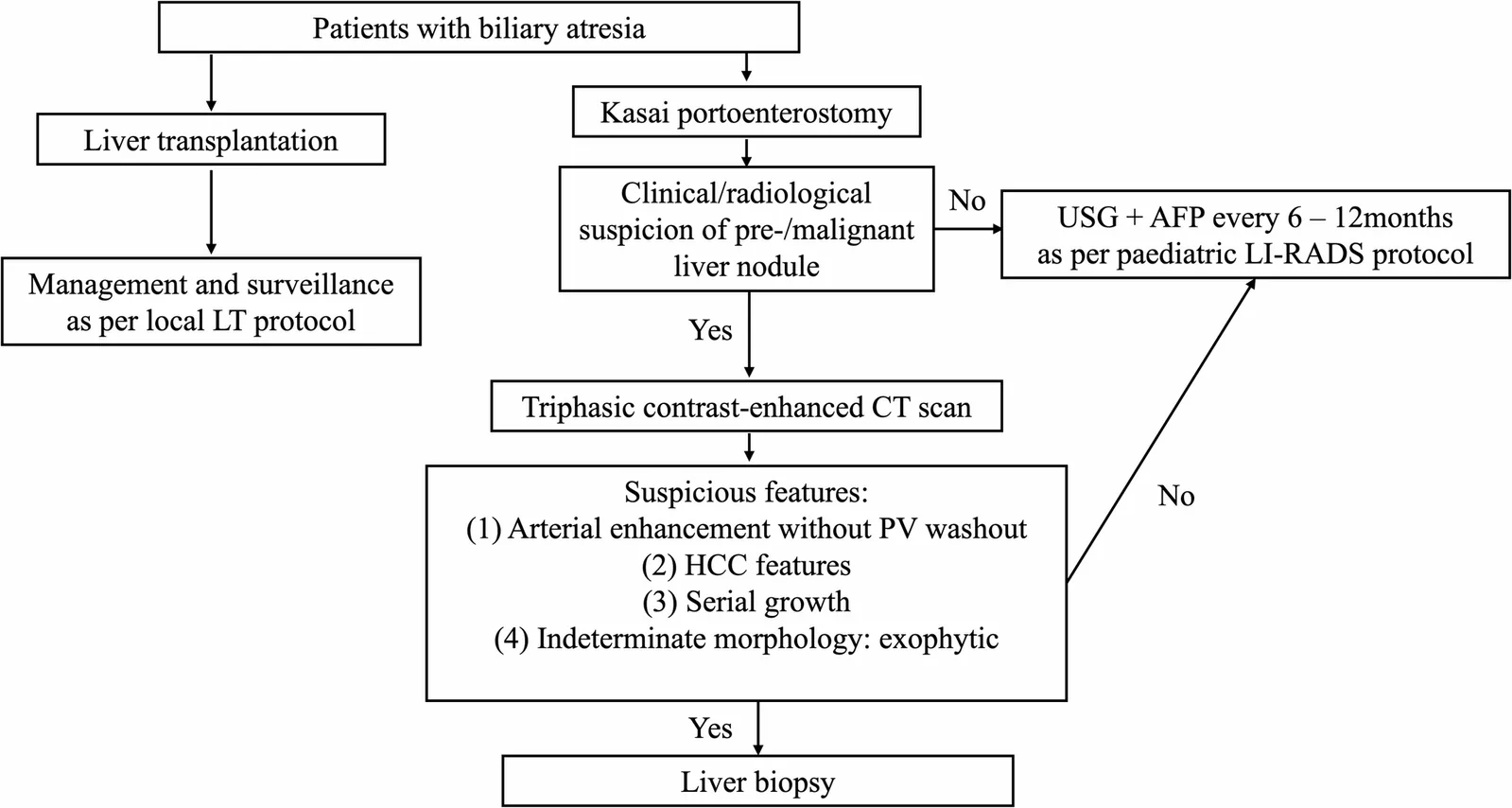
Suggested surveillance algorithm for hepatic nodules in BA NLS. *LT *liver transplantation, *USG *ultrasonography, *CT *computed tomography, *AFP *alpha-fetoprotein, *PV *portovenous, *HCC *hepatocellular carcinoma, *LI-RADS *Liver Imaging Reporting and Data System

In conclusion, approximately one-quarter of native liver survivors after KPE develop hepatic nodules. Although malignant nodules were not detected at diagnosis, dysplastic changes occur and one progressed to early HCC. None of the currently available serum tumour markers (AFP, CEA) reliably discriminated dysplastic from benign lesions, and routine liver biochemistry was equally unhelpful. On the other hand, contrast-enhanced CT consistently identified dysplastic nodules by their arterial phase enhancement without definite portovenous washout. Therefore, our observations suggest that short-term surveillance with contrast-enhanced CT may be valuable regardless of tumour marker levels, but this strategy indeed requires further validation in larger cohorts or randomized controlled studies. Nevertheless, our preliminarily suggested surveillance strategy may help reduce unnecessary invasive procedures while enabling early detection of potentially premalignant changes in this growing and vulnerable population (Fig. 6).

## Data Availability

No datasets were generated or analysed during the current study.
